# Daily fluctuations in blood glucose with normal aging are inversely related to hippocampal synaptic mitochondrial proteins

**DOI:** 10.1016/j.nbas.2024.100116

**Published:** 2024-04-05

**Authors:** Paul W. Braunstein, David J. Horovitz, Andreina M. Hampton, Fiona Hollis, Lori A. Newman, Reilly T. Enos, Joseph A. McQuail

**Affiliations:** a Department of Pharmacology, Physiology, and Neuroscience, University of South Carolina School of Medicine, Columbia, SC, USA; b Department of Neuroscience, University of Florida, Gainesville, FL, USA; c Department of Psychological Science, Vassar College, Poughkeepsie, NY, USA; d Department of Pathology, Microbiology, and Immunology, University of South Carolina School of Medicine, Columbia, SC, USA

**Keywords:** Blood glucose, Normal aging, Hippocampus, Oxidative phosphorylation, Glucose transport, Synapse, BDNF

## Abstract

Defective brain glucose utilization is a hallmark of Alzheimer’s disease (AD) while Type II diabetes and elevated blood glucose escalate the risk for AD in later life. Isolating contributions of normal aging from coincident metabolic or brain diseases could lead to refined approaches to manage specific health risks and optimize treatments targeted to susceptible older individuals. We evaluated metabolic, neuroendocrine, and neurobiological differences between young adult (6 months) and aged (24 months) male rats. Compared to young adults, blood glucose was significantly greater in aged rats at the start of the dark phase of the day but not during the light phase. When challenged with physical restraint, a potent stressor, aged rats effected no change in blood glucose whereas blood glucose increased in young adults. Tissues were evaluated for markers of oxidative phosphorylation (OXPHOS), neuronal glucose transport, and synapses. Outright differences in protein levels between age groups were not evident, but circadian blood glucose was inversely related to OXPHOS proteins in hippocampal synaptosomes, independent of age. The neuronal glucose transporter, GLUT3, was positively associated with circadian blood glucose in young adults whereas aged rats tended to show the opposite trend. Our data demonstrate aging increases daily fluctuations in blood glucose and, at the level of individual differences, negatively associates with proteins related to synaptic OXPHOS. Our findings imply that glucose dyshomeostasis may exacerbate metabolic aspects of synaptic dysfunction that contribute to risk for age-related brain disorders.

## 1 Introduction

Type II diabetes mellitus (T2DM) is an age-associated metabolic disorder characterized by dysregulated blood glucose that increases risk for mild cognitive impairment (MCI), Alzheimer’s disease (AD), and dementia among the elderly and accelerates progression from MCI to AD [1], [2], [3], [4], [5], [6]. However, even among cognitively normal older individuals, diabetes is associated with reliable, albeit modest, reductions in brain glucose uptake [7]. Moreover, subtly elevated blood glucose, that remains below the threshold for diabetes, predicts greater hippocampal atrophy among older humans [8]. Age reliably correlates with decreased glucose tolerance, increased blood glucose, and insulin resistance [9], [10], [11], [12]. While T2DM and AD are age-related diseases with significant comorbidity, there is an incomplete understanding for the basis by which changes to blood glucose that arise during normal physiological aging contribute to escalating risk for metabolic and brain disorder.

Synaptic neurotransmission is a major driver of brain energy demand [13], [14], [15], so changes at the level of synapses are credibly poised to connect age-related differences in blood glucose to neurobiological sequelae that hasten decline towards brain disorder. Consistent with this view, synapse loss is an early and reliable indicator of cognitive function among patients with MCI and AD, more so than neuropathological changes [16], [17], [18], [19], [20], [21]. Furthermore, AD-related synapse loss is strongly correlated with lower glucose utilization [22] and levels of the chief neuronal glucose transporter, GLUT3, are significantly lower in the brains of AD patients [23], [24]. Deficiencies at synapses and in neuronal glucose utilization of the AD brain maybe attributable to inefficient mitochondrial oxidative phosphorylation (OXPHOS) as post-mortem studies imply mitochondria localized to synaptic terminals are particularly affected [25]. Comparatively fewer studies investigate parallel changes over the lifespan, separated from AD, so the degree to which age-associated synaptic alterations, diminished neuronal glucose utilization, and synaptic mitochondrial dysfunction formally co-emerge with differences in blood glucose is uncertain. As such, the goals of this study were 3-fold. First, we aimed to characterize blood glucose and associated metabolic measurements in a preclinical model of normal (i.e., non-transgenic, non-pathological) aging in the rat. Second, in brain tissue obtained from physiologically characterized young adult and aged rats, we assessed whether proteins localized to synaptic terminals and associated with mitochondrial oxidative phosphorylation were correlated with individual differences in blood glucose. Third, we investigated relationships between blood glucose and protein levels of synaptophysin (SYP), a synaptic marker, neuronal glucose transporter GLUT3, and brain derived neurotrophic factor (BDNF) in the hippocampus.

## 2 Methods and materials

### 2.1 Subjects

Male, Fischer 344 × Brown Norway F1 (FBNF1) hybrid rats were acquired from the National Institute on Aging (NIA) Aged Rodent Colony (maintained by Charles River Laboratories) at 6 (n = 16) or 24 months (mo.; n = 16) of age. Only males were used in this study because age- and strain-matched females were not available from the NIA Colony for study by the extramural research community at the time these experiments were performed. Rats were single-housed in standard polycarbonate cages and maintained on a reversed 12-h light/dark schedule (lights off at 0800 h) and permitted ad libitum access to water and rat chow (Teklad Global 18 % Protein Rodent Diet 2018, Envigo, Madison, WI). All experimental procedures conformed to National Institutes of Health guidelines and were reviewed and approved by the Institutional Animal Care and Use Committee at the University of Florida.

#### 2.1.1 Food intake

Pre-weighed amounts of food were given to all rats at 0800 h and the amount of food that remained was weighed at 2000 h and at 0800 h (of the next day). The difference in weight from 0800 h to 2000 h was calculated to determine the amount of food consumed during the dark phase while the change in weight from 2000 h to 0800 h of the next day was calculated to determine the amount of food consumed during the light phase. Grams of food consumed were converted to kcal by multiplying by the energy density of the diet (3.1 kcal/g).

#### 2.1.2 Blood glucose and plasma corticosterone

One week after measurement of food intake, blood was collected from the lateral tail vein of all rats to analyze levels of blood glucose and plasma corticosterone (CORT) at 2100 h (1 h into the start of the light phase) and 0900 h (12 h later after the start of the next dark phase). Immediately following the 0900 h collection, rats were placed into Plexiglas restrainers for 60 min. Blood was collected after 60 min before rats were returned to home cages with additional blood collections at 120 and 180 min. At each time-point, blood glucose was first measured using the Precision Xtra Blood Glucose Monitoring System (Abbott Laboratories, Abbott Park, IL, USA). Then, up to 50 µl of blood was collected into Microvette® CB 300 K2EDTA-coated capillary tubes (16.444.100; Sarstedt, Newton, NC, USA). Whole blood was stored on ice until centrifuged at 3000 g for 10 min at + 4 °C; plasma was retained and stored at −80 °C. Plasma corticosterone (CORT) concentration was assayed in duplicate using a Corticosterone ELISA kit (ADI-901–097; ENZO Life Sciences, Farmingdale, NY, USA) according to the manufacturer’s protocol. Absorbance at 405 nm was measured using a Synergy 2.0 plate reader (BioTek, Winooski, VT, USA) connected to a computer running Gen5 software (version 1.05, BioTek). Every plate included standardized reactions prepared from known concentrations of CORT (32–20,000 pg/ml). For each standard curve, log-transformed data were fitted to a non-linear, variable slope equation using Prism software (version 8; GraphPad Software, San Diego, CA, USA) and the goodness of fit (r2) for all experiments was 0.998 or greater. The concentration of unknown samples was determined by interpolating raw absorbances to the standard curve.

### 2.2 Tissue preparation and Western blotting

One week after blood collection, all rats were fasted overnight before euthanasia by rapid decapitation. The brain was rapidly extracted from the skull and hippocampus, prefrontal cortex, and cerebellum were dissected away from surrounding tissues on an ice-cold plate and frozen on dry ice. Frozen tissues from the left hemisphere were used to prepare crude synaptosomal fractions. Tissues were weighed and homogenized in 1 ml/100 mg of tissue of tris-buffered sucrose solution (1.28 M sucrose, 4 mM EDTA, 20 mM Tris, pH 7.4) supplemented with Halt™ Protease Inhibitor Cocktail (ThermoFisher Scientific), then centrifuged at 1,000 g for 10 min at 4 °C to pellet nuclei and cellular debris. The supernatant, which contained synaptosomes, was transferred into a separate microcentrifuge tube. Frozen hippocampi from the right hemisphere were weighed and homogenized in 1 ml/100 mg of tissue of ice-cold RIPA buffer (0.5 % (w/v) sodium deoxycholate, 0.1 % (w/v) Triton X-100, 0.1 % (w/v) sodium dodecyl sulfate, 150 mM NaCl, and 50 mM Tris-HCl, pH 8.0) with protease inhibitors. Insoluble material was sedimented by centrifugation at 21,964 g for 20 min at 4 °C. Protein concentration of all hippocampal extracts was determined using the Pierce™ BCA Protein Assay Kit (ThermoFisher Scientific).

**Fig. 1:**
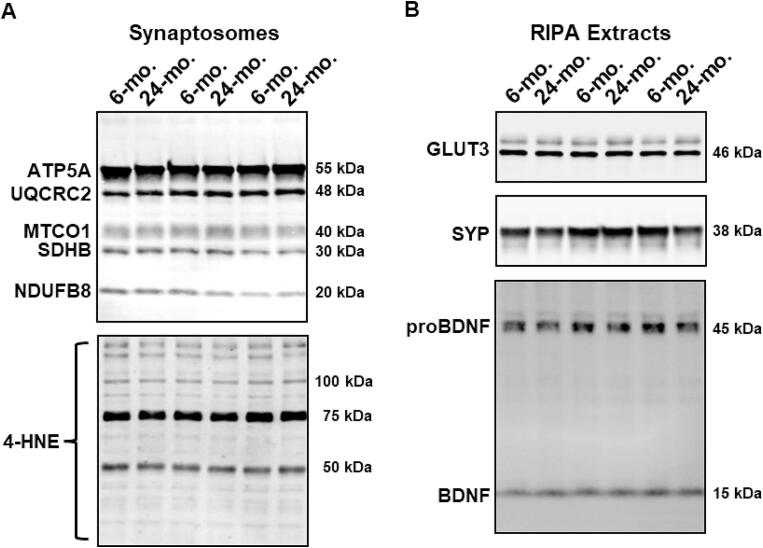
**Representative immunoreactive bands in homogenates prepared from hippocampus of young or aged male FBNF1 hybrid rats. A:** Representative immunoreactive bands detected with Total OXPHOS antibody cocktail (top) or anti-4-HNE antibody (bottom) in hippocampal synaptic terminals from 6-months old (6-mo.) and 24-months old (24-mo.) rats. **B:** Representative immunoreactive bands detected with anti-GLUT3 (top), anti-SYP (middle), and anti-BDNF (bottom) in RIPA-soluble extracts. For all images, six independent biological replicates (3/age) were selected from a representative experiment to prepare this image. Acquired images were then imported into ImageJ using the BioFormats Plug-in; brightness and contrast were adjusted equally. The final panel was organized/annotated using Microsoft PowerPoint.

Western blotting was used to measure protein levels of representative subunits from each of the five complexes that form the electron transport chain in synaptosomal fractions as well as 4-hydroxynonenal (4-HNE), a marker of oxidative stress. To contextualize changes localized to synaptosomes, RIPA-soluble fractions were also prepared to measure levels of SYP, a presynaptic vesicle glycoprotein commonly used as a marker of synapse loss in aging [17], [20], [21], [26], GLUT3, the primary neuronal glucose transporter that is sensitive to changes in blood glucose [27], and BDNF, a protein associated with impaired learning and memory following diet-induced energy excess or insulin resistance [28], [29], [30]. Nominally, 10 µg of synaptosomal or 10–20 µg of RIPA-soluble proteins were adjusted to a final volume of 10 µl in 1 × Laemmli buffer containing 5 % (v/v) beta-mercaptoethanol and loaded into wells of 4–15 % Criterion™ TGX Stain-Free™ Protein Gels (Bio-Rad Laboratories, Hercules, CA, USA). Proteins were electrophoretically separated at 200 V for 40 min in tris–glycine running buffer (Bio-Rad). Total protein staining was activated and visualized using a Bio-Rad XR + Gel Documentation System. Separated and stained proteins were transferred to low-fluorescence PVDF membranes using the Bio-Rad Trans-Blot Turbo Transfer System and total protein staining was re-imaged, post-transfer. All samples were assayed in triplicate, and the loading order of samples was randomized between gels and experiments to control for systematic variation in the electrophoresis and electroblotting procedures. Membranes were blocked for 1 h at room temperature before incubating overnight at 4 °C on a shaker with primary antibodies raised against OXPHOS subunits (Total OXPHOS Rodent WB Antibody Cocktail; ab110413 from Abcam, Waltham, MA, USA; RRID:AB_2629281), 4-HNE (AB5605 from Millipore, Burlington, MA, USA; RRID: AB_569332), SYP (ab8049 from Abcam; RRID:AB_2198854), GLUT3 (ab41525 from Abcam; RRID:AB_2736916), BDNF (ab108319 from Abcam; RRID: AB_10862052) diluted 1:1000 into blocking buffer supplemented with 0.1 % (w/v) Triton X-100. Membranes were washed in tris buffered saline before incubation in appropriate secondary antibodies conjugated to IRDye680 (diluted 1:20000; LI-COR, Lincoln, NE, USA) or IRDye800 (diluted 1:15000; LI-COR) for 1 h in the dark at 25 °C. Finally, the blots were imaged in the 700 (ex: 685 nm/em: 710–730 nm) and 800 (ex: 785 nm/em: 812–832 nm) channels of a LI-COR Odyssey CLX System. Acquired images were exported into TIF format and raw integrated intensities of immunoreactive bands were quantified using Image J (National Institutes of Health, USA; https://imagej.nih.gov/ij/). Specificity of antibodies was determined by the presence of an immunoreactive band at the predicted molecular weight relative to Precision Plus Protein All Blue Standards (10–250 kDa; Bio-Rad) included in every experimental replication (Fig. 1). Intensities of proteins of interest for each replication were normalized according to total protein to adjust for any variation in loading or transfer and transformed to the ratio of the average of all matching bands within the experiment (i.e., mean of all intensities is set to 1).

### 2.3 Statistical analysis

Data were analyzed using JASP version 0.16.4 (University of Amsterdam) and α = 0.05 for all comparisons. The Shapiro-Wilk test was used to determine which outcome variables were not normally distributed, and when appropriate, non-parametric tests (Mann-Whitney U or Spearman’s rho) were used in place of parametric analyses. Physiological measurements were compared between age groups (2 levels: 6- or 24-mo.) using independent samples *t*-test for those not assessed in a repeated manner. A 2-factor, mixed-measures ANOVA was used to examine effects of age and time for measurements repeated across the circadian cycle (2 levels: light and dark) or in response to physical restraint (4 levels: 0, 60, 120, and 180 min). The Huynh-Feldt correction was used whenever Mauchly's test indicated that the assumption of sphericity was violated. Main effects of age or interactions between age and time were followed with t-tests comparing between age groups at each time-point. Protein levels in homogenates were compared between age groups using independent samples *t*-test. Associations between blood glucose and protein levels were determined by means of bivariate correlations first conducted among samples from both age groups followed by correlations conducted separately for each age group. Circadian change in blood glucose concentration (ΔBlood Glucose = [Dark]-[Light]) was used to implement correlative analyses with proteins of interest because our statistical results (see next section) determined that age and time-of-day interacted to influence blood glucose; the difference between, rather than average across, timepoints provides a collapsed metric that reflects the outcome of the mixed between- and repeated-measures parent ANOVA. Estimation statistics were computed to determine effect size of age (Cohen’s d, rank biserial correlation) or strength of association (Pearson’s r, Spearman’s rho).

## 3 Results

### 3.1 Circadian regulation of blood glucose is impaired in aged rats and dissociated from corticosterone

**Fig. 2:**
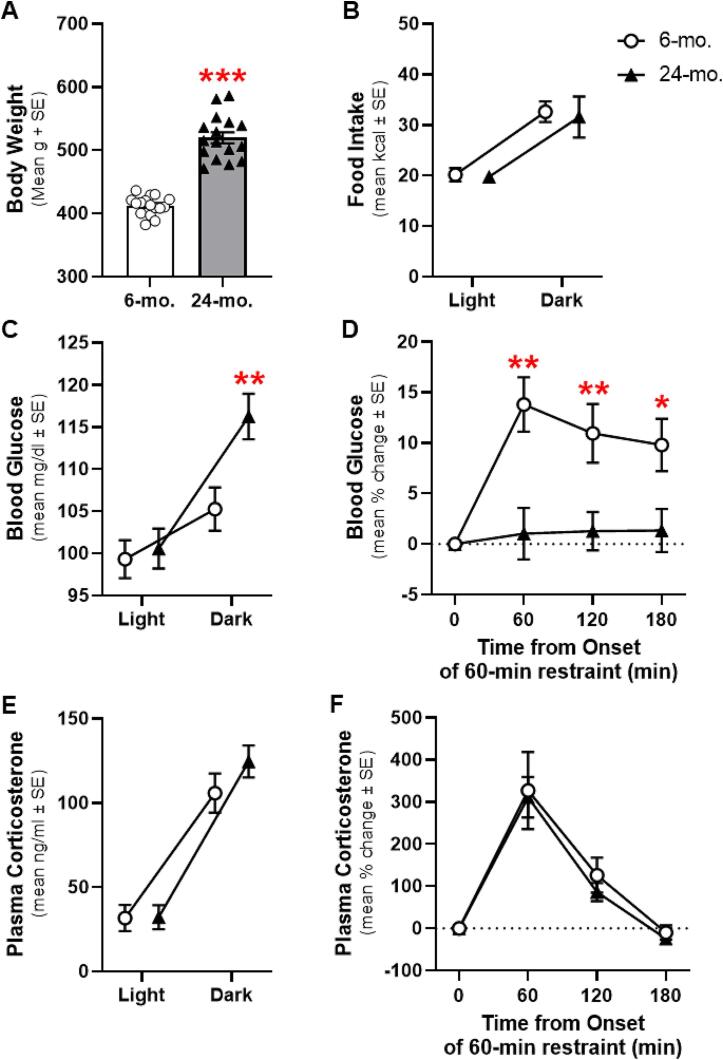
**Physiological parameters in young adult or aged male FBNF1 hybrid rats. A:** Body weight (y-axis) of 24-months old (24-mo; filled triangles and shaded column) rats is significantly greater compared to 6-mo. (open circles/column). **B:** Food intake (y-axis) is modulated between the light and dark phases of the circadian cycle (x-axis) and without any difference between age groups. **C:** Blood glucose (y-axis) is significantly different between light and dark phases of the circadian cycle (x-axis) and was greater in 24-mo. vs. 6-mo. at the beginning of the dark phase. **D:** The change in blood glucose (y-axis) stimulated by 60 min of physical restraint and during recovery (x-axis) was significantly greater in 6-mo. vs. 24-mo. **E&F:** Concentration of plasma corticosterone (y-axis; E) and change in plasma corticosterone (y-axis; F) differed with time of day (x-axis; E) or during and after recovery from restraint (x-axis; F), but did not reliably interact with age. *p < 0.05, **p < 0.01, ***p < 0.001 6-mo. vs. 24-mo.

Body weight of 24-mo. rats was significantly greater than 6-mo. rats (t(30) = 11.271, p < 0.001, d = 3.985; Fig. 2A). Differences in body weight could not be attributed to changes in food intake, as 6- and 24-mo. rats consumed a similar number of calories (F(1,30) = 0.093, p = 0.762; Fig. 2B). Circadian modulation of food intake was evident, as rats consumed more calories in the dark (main effect of time of day: F(1,30) = 23.505, p < 0.001), but this did not interact with age (F(1,30) = 0.017, p = 0.896).

Blood glucose concentration differed reliably between ages (F(1,30) = 4.775, p = 0.037) and as a function of time of day (F(1,30) = 26.443, p < 0.001; interaction with age: F(1,30) = 5.375, p = 0.027; Fig. 2C). Post hoc comparisons revealed that differences were attributed to significantly greater blood glucose of 24-mo. rats at the start of the dark phase of the day (t(30) = 2.950, p = 0.006, d = 1.043). Dysregulation of blood glucose was not accompanied by any age-related disruption to plasma corticosterone (F(1,30) = 0.819p = 0.373), though levels of this hormone also increased between the light and dark phases of the day in an age-independent fashion (main effect of time of day: F(1,30) = 122.360, p < 0.001; interaction with age: F(1,30) = 1.456, p = 0.237; Fig. 2E).

As a further challenge to increase energy demand, all rats were subjected to 60 min of physical restraint stress. This challenge produced a significant increase in blood glucose (main effect of time: F(2.447, 73.414) = 7.549, p < 0.001), that was reliably attenuated in 24-mo. rats at all timepoints (main effect of age: F(1,30) = 12.040, p = 0.002; age-by-time interaction: F(2.447, 73.414) = 5.234, p = 0.005; ts(30) = 2.524–3.452, ps < 0.017 at 60, 120, and 180 min; Fig. 2D). Similar to circadian variation, these differences in blood glucose were selective as contemporaneous changes in plasma corticosterone between 6- and 24-mo. rats were comparable (main effect of age: F(1,30) = 0.191, p = 0.665; main effect of time: F(1.192, 35.751) = 41.895, p < 0.001; age-by-time interaction: F(1.192,35.751) = 0.119, p = 0.776; Fig. 2F). Notably, elevation of blood glucose persisted for at least 120 min after release from physical restraint whereas plasma corticosterone returned to pre-stress levels over the same interval.

### 3.2 Higher variation in blood glucose is negatively associated with OXPHOS protein subunits in hippocampal synaptic terminals

**Fig. 3:**
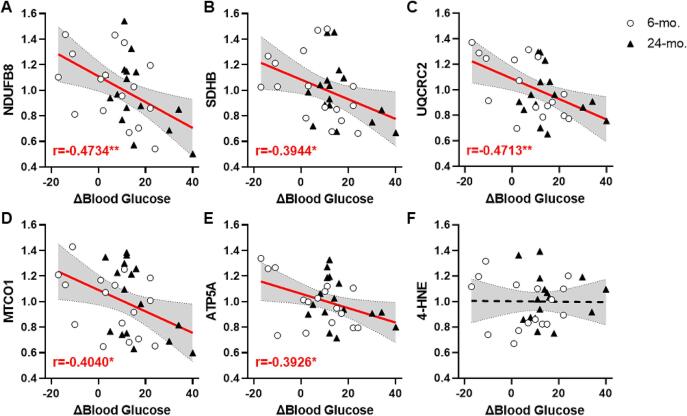
**Correlations between blood glucose and OXPHOS subunits in hippocampal synaptic terminals of aging male FBNF1 hybrid rats. A-E:** There was a significant inverse correlation between blood glucose (x-axis) and levels of OXPHOS subunits (y-axis) of ETC Complexes I-V including NDFUB8 (B), SDHB (C), UQCRC2 (D), MTCO1 (E), and ATP5A (F) in hippocampal synaptic terminals isolated from 6-mo. (open circles) and 24-mo (filled triangles) rats. **F:** There was no reliable relationship between blood glucose and 4-HNE. The line of best fit is plotted and bounded with 95 % confidence interval. Bold lines plotted in red denote p < 0.05. Inset: Pearson’s r, **p < 0.05, **p < 0.01.

OXPHOS subunits in hippocampal synaptic terminals were not significantly different between 24-mo. and 6-mo. rats (ps > 0.5; Table S1). However, bivariate correlations that tested for association with circadian change in blood glucose concentration (ΔBlood Glucose = [Dark]-[Light]) revealed reliable, inverse associations with all OXPHOS subunits measured in 6- and 24-mo. rats (NDUFB8: r = -0.473, p = 0.006; SDHB: r = -0.394, p = 0.025; UQCRC2: r = -0.471, p = 0.006; MTCO1: r = -0.404, p = 0.022; ATP5A: r = -0.393, p = 0.026; Fig. 3A-E; Table S2). Only UQCRC2 was positively correlated with blood glucose in 6-mo. rats alone (r = -0.61, p = 0.012); no proteins reliably correlated with blood glucose specifically in 24-mo. rats. However, NDUFB8 trended towards negative associations with blood glucose of both 6-mo. (r = -0.429, p = 0.097) and 24-mo. rats (ρ = -0.44, p = 0.088). Associations between circadian change in blood glucose and synaptic OXPHOS appear specific to hippocampus as these effects did not generalize to the prefrontal cortex (Tables S5 and S6) or the cerebellum (Tables S7 and S8). Further there was no effect of age (t(30) = -0.764, p > 0.4; Table S1), or association with circadian change in blood glucose (r = -0.035, p = 0.850; Fig. 3F; Table S2), on levels of 4-HNE-modified synaptosomal proteins in hippocampus.

### 3.3 Blood glucose is positively correlated with SYP and GLUT3 in hippocampus of young adult rats

**Fig. 4:**
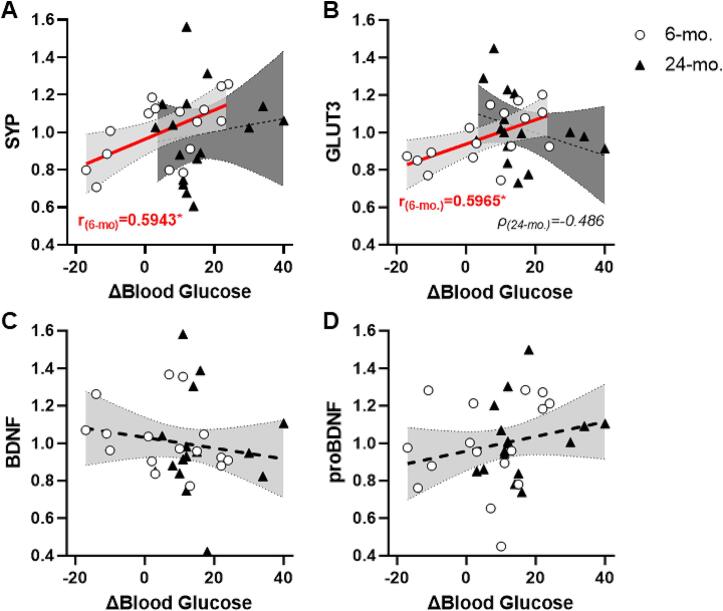
**Correlations among blood glucose, SYP, GLUT3, and BDNF in young adult or aged male FBNF1 hybrid rats. A:** Blood glucose (x-axis) of 6-months old (6-mo; open circles) rats is positively associated with hippocampal SYP protein (y-axis) whereas there is no reliable association in 24-mo rats (filled triangles). **B:** Blood glucose (x-axis) of 6-months old (6-mo; open circles) rats is positively associated with hippocampal GLUT3 protein whereas there is a marginal (p = 0.057) inverse association in 24-mo rats. **C&D:** There is no association between blood glucose and BDNF or proBDNF. The line of best fit is plotted for and bounded with 95 % confidence interval. *Bold lines plotted in red denote p < 0.05. Inset: Pearson’s r or Spearman’s rho for significant or marginal associations.

There was no effect of age on hippocampal levels of SYP (t(30) = 0.248, p > 0.8) or GLUT3 (t(30) = -0.796, ps > 0.4; Table S3). However, within-age group bivariate correlations that assessed association with circadian change in blood glucose revealed reliable, positive relationships in 6-mo. rats with both SYP (r = 0.594, p = 0.015; Fig. 4A) and GLUT3 (r = 0.560, p = 0.024; Fig. 4B; Table S4). By contrast, there were no relationships between circadian change in blood glucose and SYP (r = 0.138, p > 0.6) in 24-mo. rats and merely a trend towards a negative relationship with GLUT3 (rho = -0.486, p = 0.057; Fig. 4B). Lastly, there was no effect of age on level of BDNF (MWU = 147, p > 0.4) or proBDNF (t(30) = -0.394, p > 0.6; Table S3) nor any reliable correlation with blood glucose (rs < 0.3, ps > 0.2; Fig. 4C&D; Table S4).

## 4 Discussion

Defects in glucose utilization are a defining hallmark of the AD brain while age-associated metabolic disorders such as diabetes and obesity, which present with marked dysregulation of glucose, significantly escalate risk for AD or severity of AD symptoms in later life [1], [2], [3], [4], [5], [6]. Given these multi-directional interactions, distinguishing normal aging from central and peripheral diseases that become more prevalent with age could lead to improved approaches to identify and manage specific health risks and optimize treatments targeted to defined subpopulations of the at-risk elderly. The present study assessed individual differences in daily fluctuations of blood glucose and hippocampal OXPHOS, synapses, or neuronal glucose transport in a rat model of non-pathological brain aging. These data connect changes in energy homeostasis to synaptic decrements in susceptible, normally aging individuals and position age-related changes in blood glucose and associations with markers of neuronal and synaptic function to interact with chronic metabolic disorders or incipient brain disease to further perturb neural function.

### 4.1 Normal aging and peripheral metabolism

A key finding of our study is greater circadian variation in blood glucose of normally aging, male rats. We determined that blood glucose concentrations were significantly elevated during the nocturnal (active) period in aged rats compared to young adults, and this excess blood glucose precluded any further increase when challenged with an acute and inescapable stressor. Our data highlight that blood glucose levels are dynamic, and interpretations derived from assessments at single time points or after fasting may affect conclusions about the influence of aging on this critical metabolic parameter. Here we measured blood glucose in freely feeding rats and revealed age-related differences across periods of rest, activity, and acute stress, which contrasts with at least one other study that found no effect of age on blood glucose when measured once in fasted, FBNF1 hybrid rats [31]. However, our findings do agree with those of Masoro and colleagues [32] who uncovered age-related differences of circadian blood glucose in aging F344 rats while others found no changes when only sampling blood at single time points during the light phase [33], [34], [35]. The present and published data are also aligned with impaired glucose tolerance of male F344 rats at 26–27 months of age [36], [37].

These alterations to blood glucose in a rat model of normal aging offer interesting parallels and notable differences from rodent models of diabetes. Not surprisingly, changes to blood glucose in aged male rats co-occurred with greater body weight. Age-related weight gain in FBNF1 male rats is attributable to increasing body fat but no change of lean mass [31], [38]. This observation is consequential to the degree that body composition influences, or is influenced by, parallel physiological processes that include age-related changes in levels of, and sensitivity to, leptin and insulin that also modulate blood glucose levels [31], [39], [40]. While leptin’s suppressive effect on feeding declines with age, hormone actions are reinstated by calorie restriction that also produces weight loss [41]. Leptin receptor-deficient db/db mice are a common model of Type II diabetes, developing hyperglycemia and obesity as well as hyperphagia [42]. Elimination of insulin-producing cells with streptozotocin (STZ) also induces hyperglycemia in rodents, but significant weight loss ensues despite over-eating [43]. While rats used in the present study were not calorie restricted, it is notable that food intake was similar between lighter, young adult male rats and heavier, aged male rats and remained comparably sensitive to modulation by the circadian cycle, which agrees with longitudinal data showing aging does not change daily food intake [44]. Thus, our data confirm that the phenotype of naturally aging male rats is characterized by elevated blood glucose during the active phase and significantly greater body weight, relative to young adults, but without gross differences in food intake, which notably diverges from hyperphagia that is evident in the db/db or STZ diabetic models.

It is also noteworthy that observed differences in regulation of blood glucose were not accompanied by age-related changes to circadian or stress-related secretion of CORT. Indeed, our finding of similar CORT responses between young adult and aged male FBNF1 rats during and following restraint stress agrees with that of Segar and colleagues [45]. With respect to blood glucose changes in response to an acute stressor, Silverstein and colleagues [46] found no differences following restraint stress between male F344 rats of 7, 16, or 23 months, whereas Odio and Brodish found significantly smaller increases in blood glucose of 22–26 months-old male F344 rats compared to younger controls after exposure to motion or foot-shock stress [47], [48]. As such, the deficits observed in our study and others may relate to impairment of gluconeogenesis and not glycogenolysis as Hernandez and colleagues [39] demonstrated that aging to 24 months does not alter epinephrine-stimulated secretion of glucose from the liver in male hybrid rats.

### 4.2 Associations of blood glucose with synaptic OXPHOS

Our findings also reveal a modest, but reliable, inverse association between individual differences in circadian blood glucose and OXPHOS subunits localized to hippocampal synaptic terminals. The status of mitochondria that localize to synaptic terminals is of interest given their vital role in support of neurotransmission and their hypothesized susceptibility in aging and related brain disorders [16], [17], [18], [19], [20], [21]. Expression of genes that encode for subunits of Complexes I-V of the electron transport chain (ETC) are reliably lower in the brain of AD patients compared to age-matched normal controls [49], [50]. Changes in gene expression coincide with lower OXPHOS protein content as well as structural abnormalities of hippocampal mitochondria in AD [51], [52]. Deficits in hippocampal OXPHOS protein levels and ETC function are mimicked by over-expression of human mutant amyloid precursor protein (APP) in mice [53], [54], [55]. Using a similar OXPHOS antibody cocktail as in the present study, one study reported lower protein content of Complex I subunit NDUFB8 and Complex IV subunit II MTCO2 in normally aging mice, but significantly greater level of Complex V subunit ATP synthase subunit alpha and no changes to Complex II subunit 30 kDa SDHB or Complex III subunit Core 2 UQCRC2 [56]. Lower levels of subunits that form ETC Complexes I and IV complement other data reporting lower enzyme activity of those complexes [57], [58], [59], [60]. Mindful that contributions of Complexes I and IV may be especially consequential to brain aging, it bears mention that analyzing associations of blood glucose with OXPHOS subunits separately within age groups reduced the significance of all associations whereas the effect sizes of NDUFB8 and MTCO1 were largely unaffected, continuing to reflect medium-sized effects (i.e., r > 0.4) for both proteins in either age group. By contrast, splitting by age clearly reduced effect sizes for other OXPHOS subunits in the aged group (i.e., r < 0.3) and, in fact, strongly implicate that any possible overall association between blood glucose and UQCRC2 is driven by a relationship that is specific to young adults and without any association in aged hippocampus. Furthermore, synaptic OXPHOS in hippocampus may be comparatively more sensitive to fluctuations in blood glucose as parallel studies of prefrontal cortex and cerebellum in the same animals did not uncover evidence for effects of age or associations with blood glucose.

Critically, ETC output is not uniformly affected by aging, rather ATP production is suppressed whereas production of reactive oxygen species (ROS) is enhanced [57], [61]. This divergent influence of aging on ETC activity may explain equivocal reports of unchanged or enhanced mitochondrial oxygen consumption but lower ATP production in the brains of diabetic db/db mice [53], [62], [63]. Such imbalances jointly suggest age- and diabetes-related changes may be consequences of accumulating ROS or compensatory to prevent further ROS damage in synapses [20]. In the present study there was no effect of age on abundance of 4-HNE-modified proteins in hippocampal synaptic terminals, nor was there an association with blood glucose. 4-HNE is a by-product of lipid peroxidation and an indirect measure of oxidative stress, but in isolation, the lack of any effect should not be interpreted as definitive when other modifications (e.g., carbonylation), molecules (e.g., H2O2), enzymes (e.g., superoxide dismutase) and antioxidants (e.g., glutathione) collectively regulate ROS. Mindful of these findings, and that properties of mitochondria vary among hippocampal subregions, between subcellular compartments, and with advancing age [61], [64] our data highlight that individual differences in blood glucose, not just advanced age, reliably predict hippocampal synaptic OXPHOS over the full lifespan and further studies of mitochondrial outputs are warranted.

### 4.3 Associations of blood glucose with markers of synaptic vesicles, neuronal glucose transport, and learning & memory

As a follow-up to characterize the context in which observed changes to hippocampal synaptic OXPHOS emerge, we also determined that normal aging uncouples blood glucose from hippocampal levels of the essential synaptic vesicle-associated protein, SYP, and the neuronal glucose transporter, GLUT3. More specifically, there is a positive, linear relationship between changes in daily blood glucose and SYP in young adult rats, but in aging rats, there is no reliable relationship between these two parameters. Similarly, GLUT3 is positively correlated with circadian changes in blood glucose in young adult rats while associations in aged rats exhibit a negative trend. Glucose uptake is critical to synaptic function so reliable correlations between blood glucose and SYP or GLUT3 in young adults may reflect this optimal relationship. The reduction in the strength of these associations, or even the tendency towards negative relationships, among aged rats could reflect either diminished sensitivity of aging neurons to fluctuations in blood glucose or adaptations to protect the aging brain from deleterious effects of elevated blood glucose.

There is unambiguous evidence for frank loss of SYP and synapses from the hippocampus in AD, which becomes more pronounced as patients exhibit greater clinical symptoms or neuropathology [17], [18], [19], [20], [21]. Loss of SYP from the hippocampus is reproduced by over-expression of human mutant APP in mice [65], [66], [67], [68], [69] but loss of SYP in the hippocampus of normally aging rats manifests in defined synaptic terminal zones and only among individuals with confirmed behavioral impairments [26]. Indeed, our results comport with those of Nicolle and colleagues [70] that found preserved SYP in the aging hippocampus. The maintenance of hippocampal SYP in normally aging rats stands in contrast to findings from obese and hyperglycemic db/db mice that lose SYP from the hippocampus in an age-dependent fashion [71], [72], [73]. Critically, db/db mice exhibit severe hyperglycemia much earlier in the lifespan, so the null relationship between blood glucose and hippocampal SYP in normal aging may reflect subthreshold influences arising from smaller magnitude and comparatively later onset of elevated blood glucose.

GLUT3 also declines in the AD brain [24] and the hippocampus, in particular [23], though corroborating evidence from AD mouse models and naturally aging rodents is mixed [27], [74], [75], [76], [77], [78]. Likewise, it is not clear the degree to which elevated blood glucose, alone or in concert with other factors, durably modifies GLUT3 levels in the brain. One study uncovered a very modest reduction in GLUT3 mRNA from the frontal cortex of 10 weeks-old db/db mice, but more advanced aging was not studied [79]. Using the STZ diabetic model, significant reductions in GLUT3 mRNA and protein are induced throughout cortex and hippocampus and, further, recovered in an insulin dependent manner [80], [81]. While STZ robustly increases blood glucose, the induced loss of insulin producing cells is phenotypically similar to Type I diabetes, and not Type II diabetes that is prevalent in aging or with obesity. However, feeding high-fat diets that do induce insulin resistance and weight gain in rats has led to conflicting results on GLUT3 protein in the brain [82], [83]. As such, the marginally negative association between blood glucose and hippocampal GLUT3 in aging, which contrasts sharply with the reliable, positive association in young adults, could reflect adaptive compensation to cope with moderate elevations to blood glucose.

Reliable associations between daily change in blood glucose and markers of synapses or neuronal glucose transport in hippocampi of young adults were contrasted with uniformly null effects of age and absent correlations between BDNF and blood glucose in either age group or across the entire cohort. Several studies have reported that feeding carbohydrate-enriched diets to rats, including aged rats, impairs learning and memory and hippocampal synapses through a mechanism that involves BDNF [28], [29], [30]. Plausibly, no effect on BDNF was revealed in the present study because rats were consuming a standard diet that does not induce insulin resistance and food consumption was not significantly different between young adult and aged rats. On balance, our findings and others jointly suggest that dietary factors contribute modifiable risk that exacerbates age-related metabolic, neurobiological, and cognitive declines.

### 4.4 Limitations and considerations

There are limitations to, and considerations of, our study. First, our study was restricted to male rats, so it is not known if these findings generalize to females. While parallel study of males and females would have been desirable, we could not obtain age-matched females from the same source while the National Institute on Aging sought to accumulate hybrid female rats to fulfill the ongoing NIH mandate to investigate sex as a biological variable. On the one hand, epidemiological data indicate that prevalence of T2DM is greater in men relative to women [84], enhancing the public health relevance for understanding age-related changes to blood glucose in males. On the other hand, understanding the biological basis for sex-differences in prevalence of T2DM will require studies in both sexes. So, while our data in male rodents are robustly discussed in the context of previously published studies that, likewise, overwhelmingly reflect data from male subjects, there remains a pressing need to examine effects in female subjects to establish the presence, or absence, of sex differences over the lifespan. Second, our approach was descriptive, so it is not possible to infer causal mechanisms. Nevertheless, the FBNF1 rat is a vigorous model of aging used to study effects of diet or nutritional status on body composition, physical function, neurobiology, and behavior over the lifespan [85], [86]. Consequently, our findings are foundational to appreciate changes that naturally emerge with advancing age and could interact with dietary or metabolic manipulations to influence neurobiological outcomes. What’s more, the detection of specific correlations that localize to the hippocampus is useful to direct future studies designed to elucidate the mechanistic underpinning of these relationships and their influence on hippocampus-dependent cognition that is well-established to decline with age [87]. Third, it is important to consider that relationships between blood glucose and neurobiological substrates are not strictly explained by chronological age, but rather, associations with a spectrum of individual differences manifesting across young adult and aged rats. Individual differences were evident among hybrid rats, even of the same age, despite controlling for genetic background, housing and rearing, diet and nutritional status, and many other factors that cannot be easily controlled for in human subjects or clinical studies. The expansion of circadian variation in blood glucose across both age groups enabled the detection of a negative correlation with hippocampal synaptic OXPHOS. This suggests that the inter-relationships between blood glucose and synaptic OXPHOS in the hippocampus are maintained across the lifespan and decline or downregulate in proportion with age-related increases in blood glucose over the circadian cycle. SYP and GLUT3, which were positively associated with blood glucose in young adults showed no, or potentially inverted, relationships in hippocampus of aged rats. Changes in the reliability or direction of these relationships may suggest that the effect of biological aging on hippocampal synapses and neuronal glucose transport is one of uncoupling or desensitizing of neural substrates to individual differences in blood glucose. Fourth, since age-related changes in blood glucose were most prominent as an exaggerated increase at the start of the dark phase (when rats are more active), it will be important to determine the degree to which the effect of normal aging reflects a disruption to circadian rhythmicity of homeostatic and neural functions. Indeed, aging abolishes circadian modulation of the level of the key clock protein BMAL1 and gene expression of antioxidant enzymes in the hippocampus [88]. While tissues were harvested at only a single time-point for analysis in this study, future experiments should investigate circadian fluctuations of blood glucose in temporal association with tissue-specific expression of clock genes and antioxidants which may interact with levels of OXPHOS to dictate the dynamic balance between neural energy demands vs. oxidative stress.

## 5 Conclusions

Our findings draw attention to dysregulated blood glucose that emerges naturally during normal aging and its potential influence on key metabolic substrates localized to hippocampal synapses and neurons. These changes are more modest than those observed in AD or diabetes but reliably coupled with individual differences in variation of circadian blood glucose, highlighting that normalization of glucose metabolism presents a rational strategy to protect the aging brain. Knowledge of these relationships may be leveraged to develop more precise physiological and neurobiological biomarkers with which to ascertain efficacy of anti-diabetic drugs, such as metformin, or diets, including the Mediterranean or ketogenic diets, to reduce risk for, and severity of, brain disorders in aging individuals. Most broadly, these findings accentuate the underlying influence of processes in normal aging that can contribute to, and interact with, metabolic and brain diseases that become more prevalent with advancing age.

## 6 Author agreement statement

We declare that this manuscript is original, has not been published before and is not currently being considered for publication elsewhere. We confirm that the manuscript has been read and approved by all named authors and that there are no other persons who satisfied the criteria for authorship but are not listed. We further confirm that the order of authors listed in the manuscript has been approved by all of us. We understand that the Corresponding Author is the sole contact for the Editorial process. Corresponding Author is responsible for communicating with the other authors about progress, submissions of revisions and final approval of proofs. All the authors agree with the above statement.

## Ethics approval

All experimental procedures conformed to National Institutes of Health guidelines and were reviewed and approved by the Institutional Animal Care and Use Committee at the University of Florida.

## CRediT authorship contribution statement

**Paul W. Braunstein:** Formal analysis, Investigation, Visualization, Writing – original draft, Writing – review & editing. **David J. Horovitz:** Formal analysis, Investigation. **Andreina M. Hampton:** Investigation, Writing – review & editing. **Fiona Hollis:** Resources, Writing – review & editing. **Lori A. Newman:** Formal analysis, Investigation, Validation, Writing – review & editing. **Reilly T. Enos:** Resources, Writing – review & editing. **Joseph A. McQuail:** .

## Declaration of competing interest

The authors declare that they have no known competing financial interests or personal relationships that could have appeared to influence the work reported in this paper.

## Data Availability

The datasets generated during and/or analyzed during the current study are available from the corresponding author on reasonable request.
