# Supplementary material for: Daily fluctuations in blood glucose with normal aging are inversely related to hippocampal synaptic mitochondrial proteins
Source: Aging Brain. 2024 Apr 5;5:100116. doi: 10.1016/j.nbas.2024.100116 (PMC11002859; doi:10.1016/j.nbas.2024.100116)
Supplement: Supplementary data 1 [file mmc1.pdf]

Table S1. Effects of age on OXPHOS subunit and 4-HNE-conjugated protein levels in hippocampal synaptic terminals

OXPHOS Subunit	6-mo. (n=16)	24-mo. (n=16)	SW	Student's		Mann-Whitney		Effect Size
	Mean ± SD	Mean ± SD	p	t(30)	p	U	p	
**NDUFB8**	1.027 ± 0.276	0.973 ± 0.273	0.795	0.558	0.581	---	---	0.197
**SDHB**	1.021 ± 0.263	0.979 ± 0.239	0.038	---	---	134	0.838	0.047
**UQCRC2**	1.022 ± 0.228	0.978 ± 0.207	0.02	---	---	140	0.669	0.094
**MTCO1**	0.99 ± 0.244	1.01 ± 0.291	0.024	---	---	118	0.724	-0.078
**ATP5A**	0.998 ± 0.189	1.002 ± 0.182	0.241	-0.065	0.949	---	---	-0.023
**4-HNE**	0.974 ± 0.194	1.026 ± 0.191	0.317	-0.764	0.451			-0.270

SW=Shapiro-Wilk, SD=Standard deviation, effect size is given in Cohen's d for normally distributed data and rank biserial correlation for non-normally distributed data

Table S2. Correlations between circadian change in blood glucose and OXPHOS subunit and 4-HNE-conjugated protein levels in hippocampal synaptic terminals

OXPHOS Subunit	6-mo. & 24-mo. (n=32)			6-mo. (n=16)					24-mo. (n=16)				
	SW	Pearson		SW	Pearson		Spearman		SW	Pearson		Spearman	
	p	r	p	p	r	p	rho	p	p	r	p	rho	p
**NDUFB8**	0.818	-0.473**	0.006	0.964	-0.429	0.097	---	---	0.014	---	---	-0.44	0.088
**SDHB**	0.574	-0.394*	0.025	0.079	-0.422	0.104	---	---	0.005	---	---	-0.217	0.42
**UQCRC2**	0.905	-0.471**	0.006	0.418	-0.61*	0.012	---	---	0.007	---	---	-0.142	0.601
**MTCO1**	0.601	-0.404*	0.022	0.9	-0.415	0.11	---	---	0.01	---	---	-0.41	0.114
**ATP5A**	0.255	-0.393*	0.026	0.036	---	---	-0.424	0.102	0.009	---	---	-0.283	0.287
**4-HNE**	0.909	-0.012	0.949	0.030			-0.041	0.880	0.008			0.171	0.526

SW=Shapiro-Wilk Test for Bivariate Normality, *p<0.05, **p<0.01

Table S3. Effects of age on SYP, GLUT3, and BDNF protein levels in hippocampus

Protein of Interest	6-mo. (n=16)	24-mo. (n=16)	SW	Student's		Mann-Whitney		Effect size
	Mean ± SD	Mean ± SD	p	t(30)	p	U	p	
**SYP**	1.009 ± 0.174	0.991 ± 0.249	0.557	0.248	0.806			0.088
**GLUT3**	0.976 ± 0.144	1.024 ± 0.19	0.533	-0.796	0.432			-0.282
**BDNF**	1.019 ± 0.174	0.981 ± 0.271	0.026			147	0.491	0.148
**proBDNF**	0.984 ± 0.247	1.016 ± 0.198	0.915	-0.394	0.697			-0.139

SW=Shapiro-Wilk, SD=Standard deviation, effect size is given in Cohen's d for normally distributed data and rank biserial correlation for non-normally distributed data

Table S4. Correlations among circadian change in blood glucose, hippocampal SYP, GLUT3, and BDNF

Correlation	6-mo. & 24-mo. (n=32)					6-mo. (n=16)					24-mo. (n=16)				
	SW	Pearson		Spearman		SW	Pearson		Spearman		SW	Pearson		Spearman	
	p	r	p	rho	p	p	r	p	rho	p	p	r	p	rho	p
**ΔBlood Glucose-SYP**	0.817	0.293	0.103			0.625	0.594*	0.015			0.61	0.138	0.609	---	---
**ΔBlood Glucose-GLUT3**	0.501	0.17	0.351			0.332	0.597*	0.015			0.005	---	---	-0.486	0.057
**ΔBlood Glucose-BDNF**	0.017			-0.199	0.274	0.033			-0.446	0.083	0.041			0.069	0.798
**ΔBlood Glucose-proBDNF**	0.942	0.226	0.214			0.825	0.215	0.423			0.23	0.216	0.421		

SW=Shapiro-Wilk Test for Bivariate Normality, *p<0.05

Table S5. Effects of age on OXPHOS subunit protein levels in prefrontal synaptic terminals

**OXPHOS Subunit**	**6-mo. (n=16)**	**24-mo. (n=16)**	**SW**	**Student's**		**Mann-Whitney**		**Effect Size**
	**Mean ± SD**	**Mean ± SD**	**p**	**t(30)**	**p**	**U**	**p**	
**NDUFB8**	0.975 ± 0.212	1.025 ± 0.296	0.691	-0.539	0.594	---	---	-0.191
**SDHB**	0.94 ± 0.288	1.06 ± 0.347	0.39	-1.06	0.298	---	---	-0.375
**UQCRC2**	1.008 ± 0.17	0.992 ± 0.177	0.013	---	---	136	0.78	0.063
**MTCO1**	0.971 ± 0.278	1.029 ± 0.271	0.215	-0.604	0.55	---	---	-0.214
**ATP5A**	1.039 ± 0.656	0.961 ± 0.591	0.006	---	---	136	0.78	0.063

SW=Shapiro-Wilk, SD=Standard deviation, effect size is given in Cohen's d for normally distributed data and rank biserial correlation for non-normally distributed data

Table S6. Correlations between circadian change in blood glucose and OXPHOS subunit protein levels in prefrontal synaptic terminals

**OXPHOS Subunit**	**6-mo. & 24-mo. (n=32)**			**6-mo. (n=16)**			**24-mo. (n=16)**				
	**SW**	**Pearson**		**SW**	**Pearson**		**SW**	**Pearson**		**Spearman**	
	**p**	**r**	**p**	**p**	**r**	**p**	**p**	**r**	**p**	**rho**	**p**
**NDUFB8**	0.161	0.236	0.193	0.248	0.026	0.924	0.062	0.41	0.115	---	---
**SDHB**	0.566	-0.043	0.817	0.573	-0.191	0.479	0.003	---	---	0.121	0.655
**UQCRC2**	0.131	0.093	0.611	0.53	-0.23	0.391	0.124	0.559*	0.024	---	---
**MTCO1**	0.3	-0.057	0.758	0.109	-0.023	0.933	0.019	---	---	-0.21	0.436
**ATP5A**	0.57	0.16	0.381	0.503	0.364	0.166	0.105	-0.029	0.915	---	---

SW=Shapiro-Wilk Test for Bivariate Normality, *p<0.05

Table S7. Effects of age on OXPHOS subunit protein levels in cerebellar synaptic terminals

OXPHOS Subunit	6-mo. (n=16)	24-mo. (n=16)	SW	Student's		Mann-Whitney		Effect Size
	Mean ± SD	Mean ± SD	p	t(30)	p	U	p	
NDUFB8	0.961 ± 0.277	1.039 ± 0.372	0.03	---	---	118	0.724	-0.078
SDHB	0.951 ± 0.233	1.049 ± 0.335	0.855	-0.966	0.342	---	---	-0.342
UQCRC2	0.952 ± 0.23	1.048 ± 0.39	0.071	-0.855	0.399	---	---	-0.302
MTCO1	0.918 ± 0.371	1.082 ± 0.5	0.382	-1.056	0.3	---	---	-0.373
ATP5A	0.984 ± 0.444	1.016 ± 0.455	0.034	---	---	126	0.956	-0.016

SW=Shapiro-Wilk, SD=Standard deviation, effect size is given in Cohen's d for normally distributed data and rank biserial correlation for non-normally distributed data

Table S8. Correlations between circadian change in blood glucose and OXPHOS subunit protein levels in cerebellar synaptic terminals

OXPHOS Subunit	6-mo. & 24-mo. (n=32)					6-mo. (n=16)					24-mo. (n=16)				
	SW	Pearson		Spearman		SW	Pearson		Spearman		SW	Pearson		Spearman	
	p	r	p	rho	p	p	r	p	rho	p	p	r	p	rho	p
NDUFB8	0.026	---	---	0.34	0.057	0.744	0.39	0.135	---	---	0.033	---	---	0.288	0.28
SDHB	0.634	0.183	0.317	---	---	0.853	0.103	0.703	---	---	0.007	---	---	-0.037	0.892
UQCRC2	0.035	0.189	0.301	---	---	0.582	-0.075	0.782	---	---	0.078	0.324	0.22		
MTCO1	0.38	-0.016	0.932	---	---	0.519	-0.139	0.608	---		0.008	---	---	-0.134	0.62
ATP5A	0.088	-0.272	0.133	---	---	0.627	-0.429	0.097	---	---	0.053	-0.168	0.535		

SW=Shapiro-Wilk Test for Bivariate Normality
